# Disease transmission promotes evolution of host spatial patterns

**DOI:** 10.1098/rsif.2016.0463

**Published:** 2016-09

**Authors:** Michael A. Irvine, James C. Bull, Matthew J. Keeling

**Affiliations:** 1 Centre for Complexity Science, University of Warwick, Coventry, UK; 2 Mathematics Institute and Department of Biological Sciences, University of Warwick, Coventry, UK; 3 Department of Biosciences, University of Swansea, Swansea, UK

**Keywords:** pattern formation, spatial ecology, evolutionarily stable strategy, host–pathogen dynamics

## Abstract

Ecological dynamics can produce a variety of striking patterns. On ecological time scales, pattern formation has been hypothesized to be due to the interaction between a species and its local environment. On longer time scales, evolutionary factors must be taken into account. To examine the evolutionary robustness of spatial pattern formation, we construct a spatially explicit model of vegetation in the presence of a pathogen. Initially, we compare the dynamics for vegetation parameters that lead to competition induced spatial patterns and those that do not. Over ecological time scales, banded spatial patterns dramatically reduced the ability of the pathogen to spread, lowered its endemic density and hence increased the persistence of the vegetation. To gain an evolutionary understanding, each plant was given a heritable trait defining its resilience to competition; greater competition leads to lower vegetation density but stronger spatial patterns. When a disease is introduced, the selective pressure on the plant's resilience to the competition parameter is determined by the transmission of the disease. For high transmission, vegetation that has low resilience to competition and hence strong spatial patterning is an evolutionarily stable strategy. This demonstrates a novel mechanism by which striking spatial patterns can be maintained by disease-driven selection.

## 1. Introduction

Various diverse plant communities produce a range of striking regular spatial patterns, including spots, stripes and labyrinths [1]. A number of mechanistic explanations have been proposed for the occurrence of these patterns, many involving spatial plant competition mediated by the environment such as competition for nutrients or water [2–5]. These spatial structures can increase the density of vegetation locally as well as sustain a community that would otherwise be barren under homogeneous (non-spatial) assumptions [6]. It remains an open problem how such forms of spatial pattern help to regulate other processes, such as disease spread [7]. There is also a question as to how interspecific interaction influences the resulting spatial pattern and how pattern formation is maintained on evolutionary time scales [8].

Hosts and their parasites form a system where the resulting spatial pattern of either the host or parasite is subject to strong evolutionary pressures. For example, fragmentation of its host population places severe constraints on the ability of the infection to spread, favouring pathogens that can persist in small isolated host populations. Additionally, parasites or infections can evolve to limit their own spread, slowing the depletion of the host species and hence increasing their long-term persistence [9]. Local clustering of hosts can lead to pathogens with low virulence, while a coevolution of both parasite and host leads to hosts with high resistance as well as parasites with low virulence [10]. Compared to a homogeneous landscape, local clustering of plants leads to rapid initial growth of infection as it invades a single dense cluster; however, clustering can reduce the invasion of an infection on longer time scales as the pathogen must spread from one cluster to another [11]. This shows that the ability of a species to distribute itself with a fragmented spatial pattern greatly affects the outcome of a disease.

Here, we explore the dynamics of host–pathogen interaction in spatial systems using a simple model, developed to yield generalizable results, but our study is primarily motivated by the case of seagrass and its major wasting disease. Seagrasses comprise approximately 60 species of marine angiosperms, with a near-global distribution [12]. They are recognized as major ecosystem service providers [13], as well as being globally threatened by coastal development, pollution and infectious disease [14]. Several species of seagrass suffer from a wasting disease that has the potential to cause massive die-back over oceanic scales. Most notably, approximately 90% of the major species of the north Atlantic, *Zostera marina*, was lost in the epidemic of the 1930s [15]. More recently, wasting disease has resulted in severe but geographically limited seagrass die back [16]. This disease has since been attributed to the marine slime mould-like protist, *Labyrinthula zosterae* [17]. The pathogen *L. zosterae* has been isolated from over 80% of seagrass populations sampled in northern European waters [18], and shown to contribute to long-term regulation of seagrass population dynamics [19]. Currently, the pathogen is seen in an endemic state in many seagrass species, with the pathogen being successfully isolated from all of 11 species tested [20]. Little is known about the triggers of epidemic outbreak, although environmental factors including water temperature, ambient light and salinity are implicated in pathogenicity [15]. This host–pathogen system, therefore, makes an excellent case study for eco-evolutionary research in a relatively simple but widespread marine system of high ecosystem value [21]. The seagrass pathogen, *L. zosterae*, causes disease by spreading through the host tissue from a focal infection point on a seagrass leaf. Transmission has been shown to occur through direct leaf-to-leaf contact, with occasional longer range transmission through drifting infected plant material [22].

Our model is also intended to capture dynamics operating in other sessile marine organisms. For example, mussels, which are known to self-organize into complex spatial patterns at the landscape level [23], are also susceptible to large-scale dieback from bacterial pathogens. Much of the research into their epidemiology comes from studies in aquaculture [24] but several pathogenic species of *Vibrio* are found in natural bivalve populations. Little is known about their transmission but survival is relatively poor outside of their hosts, or as part of biofilms [25], making this another host–pathogen system where our model is potentially relevant.

Models where diseases evolve into a critical state such that host cluster sizes are scale-free have been previously considered [26]; in contrast, here we consider the evolution of the sessile host species in response to a virulent disease. Using a Probabilistic Cellular Automata (PCA) model, we initially show that the host's resilience to competition from other hosts in the local environment determines the type of spatial pattern observed. We then use this model to test our hypothesis that if the individual's resilience to competition is a heritable trait then this factor is under strong selection in the presence of disease. This leads to dynamics where if there is no disease locally a plant is more successful if it has low mortality due to spatial competition, as this will allow its offspring to proliferate at a higher rate. Whereas if there is a strong local disease prevalence, low resilience to competition provides a way of isolating offspring from other patches that are in a diseased state, thus increasing reproductive success.

The methods and results are split into two main sections. In the first section, the impact of regular pattern formation on the dynamics of a host–pathogen system is investigated. In particular, the role of regular banding in vegetation in limiting the spread and impact of a virulent disease is explored using a PCA model. The second section of the paper focuses on the evolution and maintenance of spatial banding in the presence of a pathogen from an evolutionary viewpoint. The main hypothesis is that banding in vegetation can be viewed as an evolved trait in the sessile host species in the presence of a disease. In which case, we assess under what conditions would such an evolved trait be expected to rise and how generally does banding impact the spread and distribution of the disease.

## 2. Material and methods

### 2.1. Disease-vegetation model

**Figure 1.**
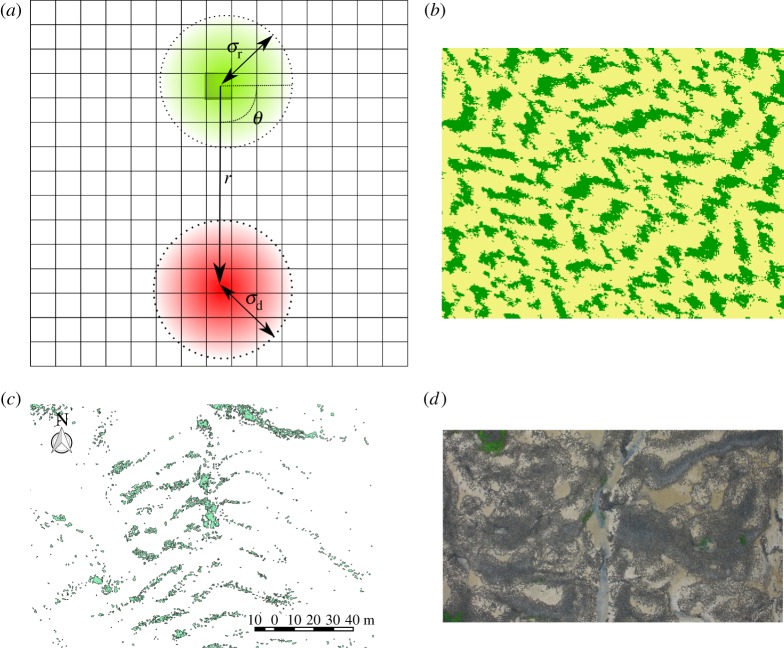
(*a*) Schematic of the regular banding model. The local reproduction kernel is shown in green with standard deviation 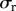. The competition kernel is shown in red, with standard deviation 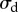 and with an offset at angle *θ* and displacement *r*. (*b*) Example of spatial distribution from a single simulation with high competition on 300 × 300 lattice with wrap-around boundary conditions (parameters: *σ*_r_ = 0.5, *σ*_d_ = 1.0, *k* = 0.1, *θ* = *π*/4, *r* = 10). Although the direction of the offset is constant throughout the whole lattice, locally the direction and strength of the banding can vary. (*c,d*) Pattern formation is ubiquitous in nature and the product of a diverse set of interactions. Examples of regular banding in two diverse ecosystems are shown here. (*c*) Seagrass banding from The Isles of Scilly, UK, with vegetation shown in green, patch borders in black and barren sand shown in white [33]. (*d*) An aerial photograph of mussels near Bangor, UK (adapted from [23]).

A PCA model was used to capture the spatial interactions between plant and disease [27,28]. This model is an extension of one that has previously been used to model the spatial evolution of seagrass [29]. It can be considered as a PCA extension to the model in [30], which is a generally applicable model of vegetation dynamics in the presence of spatial competition. The system is a 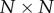 lattice, where each lattice site can be in one of three states: *E*, *O*, *D*. *E* stands for an empty site; *O* is a site occupied with healthy vegetation and *D* is a site occupied with vegetation in a diseased state. The vegetation is considered to have a primarily clonal form of growth with a local reproduction kernel *k*_r_. There is also a spatial competition process, such as competition for resources, that is mediated by a local competition kernel * k*_c_ [3,31,32]. The competition kernel has an offset such that the maximum impact of competition occurs at a distance *r* and direction *θ* due to a geographical gradient, such as prevalent wind-direction or current (figure 1). For sufficiently large offset and strength of competition, regular bands of vegetation emerge (an example of which can be seen in figure 1*b*), whereas for smaller values of competition and offset the model produces more patchy spatial patterns. Examples of both forms of pattern formation qualitatively match with observed natural patterns (figure 1*c,d*).

In addition to the vegetative growth, we also consider a disease process that has a spatial transmission scale * k*_d_ and a rate of infection *β* (noting that all rates are scaled such that the rate of local vegetative growth is one). There is also assumed to be a constant low background rate 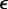 of disease importation, this was to ensure that the disease could never be completely eradicated, thus clusters of disease emerge spontaneously throughout the lattice. This importation process is biologically justified as some long-range infection events may occur that are not captured by the local spreading term. For example, in seagrass, diseased shoots can become detached and float on currents where they can come into contact with susceptible leaves [22]. The background rate was set to 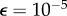 unless otherwise stated. Simulations were performed on a lattice with toroidal boundary conditions and updated synchronously. The dynamics for the competition model can be written according to the rate of a site located at **x** transitioning from state *A* to state *B* in a time-step as2.1*a*
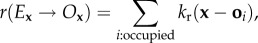
2.1*b*
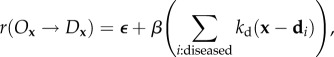
2.1*c*
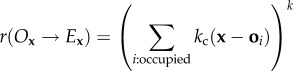
2.1*d*
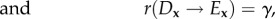
where 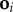 and 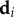 represent the location of the *i*th occupied and diseased site, respectively. The kernel *k*_r_ represents reproduction due to local clonal reproduction and takes the form of a Gaussian with zero mean and variance 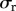. The transition from occupied site to unoccupied is due to death from competition and is mediated via the competition kernel *k*_c_, which is also Gaussian with a mean of displacement *r* and angle *θ* and a variance 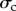. This represents death due to competition factors such as hydrological scouring and resource depletion. The mean displacement comes due to exogenous environmental factors such as gradient or prevailing current. Key to the vegetation behaviour is a dimensionless parameter *k*, which represents the resilience to competition and controls the kurtosis of the competition kernel. For low *k*, the effect of the competition is stronger. For larger *k*, the competition is weaker going to 0 as 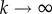. The disease propagates with a kernel *k*_d_, which is again Gaussian with zero mean and variance 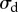. Finally, the death of an individual due to disease is assumed to occur with constant rate *γ*. As such, the expected duration of infection is 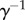.

Parameter studies were conducted on both models. Although the model has not been rigorously parametrized against data owing to the more theoretical nature of the study, certain parameters were chosen to reflect the biological details of seagrass beds. These were mean annual rhizome elongation of 26 cm yr^–1^ and *L. zosterae* spread of 1 mm h^–1^ [34,35].

### 2.2. Evolutionary model

The dominance of competition traits was explored by adapting the previous model so that each individual has its own heritable resilience value, *k*(**x**). High *k* is associated with high resilience (and limited impact of spatial competition), whereas low *k* implies low resilience and strong competitive effects. Asexual reproduction is assumed such that the offspring inherit the resilience of their associated parent. Initially, we assume that *k* can take one of two distinct values: either high or low 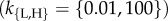 and at a birth event there is some small probability *λ* that the trait switches from low to high or vice versa by random mutation. Thus, each lattice site can be in one of four states: *E*-empty site; *L*-occupied with low resilience trait; *H*-occupied with high resilience trait; *D*-diseased.

The transition rates are defined as2.2*a*
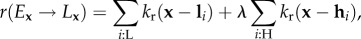
2.2*b*
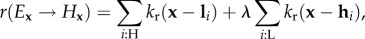
2.2*c*
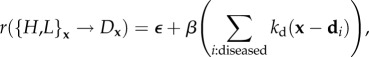
2.2*d*
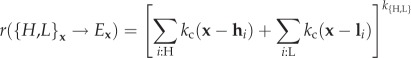
2.2*e*
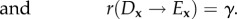


Kernels and rates are the same as the first model. Each trait has a distinct value for the dimensionless parameter given as 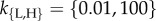. These dynamics also assume that reproduction may only occur when an individual is not infected. As we are considering a highly virulent pathogen this approximation is reasonable; however, for a low virulence pathogen, the diseased state may also need to be considered to propagate.

## 3. Results

### 3.1. Competition in regulating disease spread

**Figure 2.**
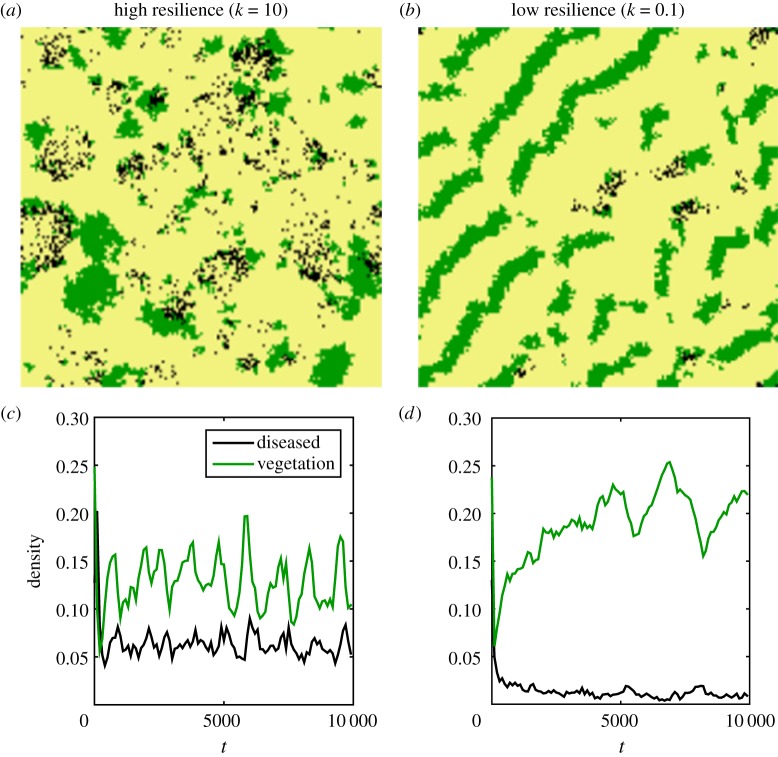
Realization of dynamics for the model described by equation (2.1) (with parameters *σ*_r_ = 0.5, *σ*_d_ = 1, *γ* = 0.2, *β* = 2) for high competition resilience (*k* = 10) (*a,c*) and low competition resilience (*k* = 0.1) (*b,d*). The figures on the top (*a,b*) show a typical snapshot of the spatial distribution of healthy vegetation (in green), the diseased state (in black) and the empty site (in yellow). The bottom figures (*c,d*) show the time series for vegetation and disease. For high competition resilience, there are constant epidemics leading to a high density of diseased vegetation and anti-persistent vegetation dynamics. Where competition resilience is low the vegetation dynamics are more persistent and the disease prevalence is far lower.

As a first investigation of how spatially distributed competition affects disease spread we compare the disease dynamics for a system which has no competition 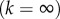, to a system where there is strong competition with offset such that banding is exhibited (*k* = 0.1) (figure 2). For a system that has no competition 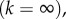 there are regular epidemics that spread throughout the population leading to a reduced density of the vegetation population with a high degree of variability and a high level of disease (figure 2*a,c*). For this no competition limit, the model is similar to the forest fire model, where the system evolves into a self-organized critical state producing a heavy-tailed patch-size distribution with patches that are frequently destroyed by diseases [36,37]. By contrast, when competition is strong (*k* = 0.1), but all other parameters are the same as the previous example, the vegetation forms into a banded structure even in the absence of disease. In this case, the disease remains endemic at far lower levels as diseased patches are constrained due to the banding structure, meaning the disease cannot continue to propagate attacking other healthy patches, thus giving previously diseased patches time to recover (figure 2*b,d*).

**Figure 3.**
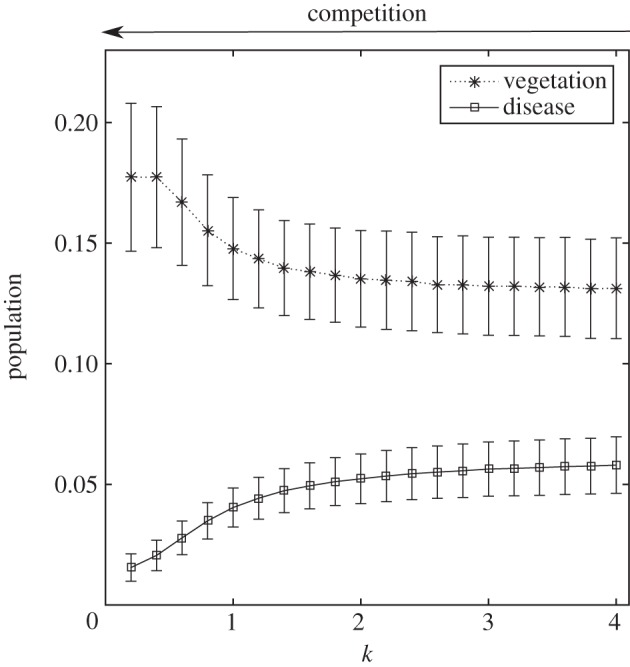
Mean and variance (shown as bars) for the population of vegetation and diseased vegetation over a range of competition resilience *k*, leaving other demographic parameters fixed at *σ*_r_ = 0.5, *σ*_c_ = *σ*_d_ = 1, *r* = 10, *θ* = *π*/4, *γ* = 0.2, *β* = 2. Increasing resilience to competition (increasing *k*) leads to lower density of healthy vegetation and higher disease prevalence. This in turn also leads to a lower variance in the disease population. Vegetation dynamics with strong competition was found to go on longer excursions than in the low competition regime where clusters quickly grow and are then invaded and quickly eradicated by disease.

In order to determine how the strength of competition affects the prevalence of disease, simulations were performed over a range of *k* values. For each 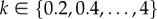, 100 replicate simulations were carried out for 10^4^ time-steps, with other parameters held constant, the disease process occurred on the same spatial scale as the competition and vegetative growth was primarily local 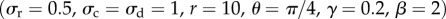. The endemic level of infection increases with *k*: competition and the spatial pattern that emerges therefore helps to regulate the incidence of endemic disease (figure 3). For low competition resilience (low *k*), bands of vegetation form that are susceptible to infection and hence death due to disease, this leads to the population performing large excursions away from the mean as large bands are infected leaving gaps that recover slowly due to the lower fecundity of the vegetation caused by the enhanced competition.

### 3.2. Evolutionary model

Simulations were performed on a 150 × 150 lattice for 10^4^ time-steps and each set of parameters simulations were repeated 100 times. For the initial conditions, 20% of sites are occupied and one half of the lattice had low resilience (*L*), while the other half had high resilience (*H*). The simulations were allowed to evolve under the dynamics described in equation (2.2).

**Figure 4.**
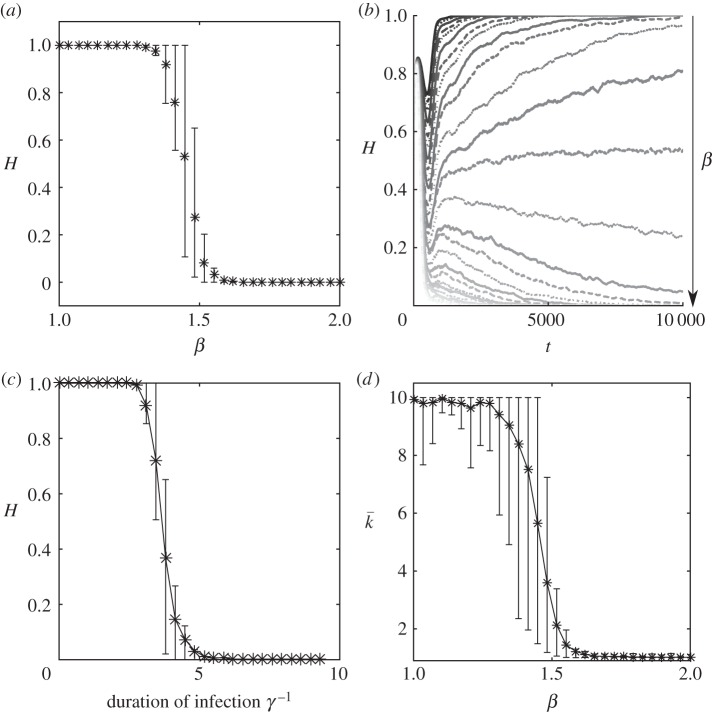
(*a*) Ensemble-averaged proportion of occupied sites in *H* state with 95 percentile values for the discrete trait model. Parameters are *γ* = 0.1, *σ*_r_ = 0.5, *σ*_c_ = 2, *σ*_d_ = 1, *λ* = 10^−7^, *r* = 2 and rate of import of infection 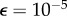. (*b*) Ensemble-averaged runs for increasing transmission. Around the critical transmission value, there is a slowing down of the time taken for the population to fixate. (*c*) Ensemble-averaged realizations of proportion of occupied sites in state *H* for the discrete trait model. Parameters are *β* = 1.7, *σ*_r_ = 0.5, *σ*_c_ = 2, *σ*_d_ = 1, *λ* = 10^−7^, *r* = 2 as *γ*^−1^ is varied between 0 and 10. (*d*) Averaged realizations, with upper and lower 95 percentiles, where the competition trait is allowed to mutate in a continuous manner. Results are broadly similar to the discrete trait model, where for low virulence high competition resilience traits dominate 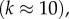 and for large virulence low resilience traits dominate 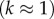. However, for intermediate values, the upper and lower percentiles show a high degree of variation between simulation runs where clusters of differing *k* values are transient.

Keeping other parameters constant and changing the value of *β*, the model displays a sharp transition from the *H* population dominating to *L* (figure 4*a*). When *β* approaches a critical point, an increase in the time for a single trait to dominate is observed. For values of *β* far away from the critical point, one strategy quickly dominates thus the relaxation time is short. Near the critical point, there is a transient coexistence of the two vegetation types. Fluctuations of the two vegetation types also increase sharply around the critical value (figure 4*b*). Population variance of the disease are high when *β* is lower than the critical point, increases sharply at the critical point and then reduces to a lower value for larger values of *β*. Population variance of the density of vegetation also peak around the critical point.

Sensitivity analysis was performed on both the duration of infectious period 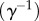 and the rate of importation of disease 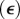. It was found that for fixed *β*, the importation rate does not alter the dominance of *L* strategy until it reaches a critical value (approx. 10^−1^ for *β* = 2), where the host population collapses. Very similar results were found for the duration of infectious period, 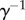 as for the transmissibility, *β*. For fixed *β* = 1.7, there is a sharp transition between *H* being the dominant strategy and *L* dominating (figure 4*c*).

To gain a greater understanding of the evolution of the resilience trait *k*, the model was further extended by allowing the strength of resilience to occupy a continuous range of values (between 0.1 and 10) as opposed to just high and low as was previously considered and to allow these values to mutate. When a mutation event occurred, a new value for *k* was drawn from a normal distribution with a mean of the parent value and a variance of one. Although biologically, the variance of the mutation is relatively high, this allowed for rapid mutation over time scales that are computationally feasible. New *k*-values were also kept in the range [13,36]. A very similar transition occurred as for the two-trait model as the virulence of the disease changed (figure 4*d*). For infection rate *β* < 1.5, the average value of the competition trait is high and thus there is little spatial competition. However, there was found to be a mixture of *k*-values as the upper and lower percentiles of the ensembles indicate. This is due to mutations arising that spread as a population and thus alter the average value of *k*. For intermediate values of virulence (1.3 < *β* < 1.6), there was found to be a large range of values the competition population averages can take. For these intermediate values, neither trait strongly out competes the other, thus leading to populations with a greater mixture of traits. For large *β*, competition trait is minimized, thus when the disease is highly transmissible banding still remains an uninvadable strategy.

## 4. Conclusion

The interaction between disease dynamics and vegetation dynamics was explored in a system with strong and weak spatial competition. The importance of competition processes within the vegetation in regulating the spread of disease was assessed by measuring the prevalence of disease over a range of strengths of competition, including those that induce banding in the vegetation. Increased competition and hence spatial banding was found to limit the prevalence of the diseased state, but increase the variance of the vegetation population due to bands of susceptible vegetation becoming infected leading to a collapse of a significant proportion of the total vegetation population. In the limit where there is no competition, the model is akin to the forest fire model [36] where the system naturally evolves into a critical state, where there can be a large cascade of epidemic events and the distribution of the size of disease outbreaks follows a power-law distribution.

Regular spatial patterns in vegetation have often been associated with environmental factors, such as nutrient levels or ground water or with biotic interactions [1,38]. These spatial patterns have been shown to provide global benefit to species, by allowing them to exist in environments that would otherwise be unfavourable and not permit their existence. Here, we have shown that as the amount of spatial competition, and thus banding increases, the prevalence of disease decreases. This would naturally lead to areas of vegetation with banding having a higher survival probability in the presence of disease, while areas of vegetation with no banding being more likely to go extinct. This shows that at the population level, one group has a higher survival probability than the other group.

The evolutionary mechanism underpinning banding in the presence of disease was explored by considering a vegetation population with two traits: high and low resilience to competition with surrounding vegetation. For small infection rates, the high resilience trait dominates and the dynamics are akin to Lotka–Volterra competition. There is a critical infection rate, however, after which the low resilience trait dominates and can be sustained even for relatively large offset of the competition. Previous work has considered pattern formation as an evolved phenotype [8]. Our unique contribution here is to demonstrate (i) how banding stabilizes a sessile community in the presence of a highly transmissible pathogen, and (ii) under what conditions do we expect banding to emerge if resilience to spatial competition is an adaptive trait.

The model considered here is one where competition is altruistic in the sense that an individual with increased competition has a higher mortality rate, thus the sacrifice of the individual in suffering greater effects of competition leads to a spatial pattern that increases the long-term success of its progeny. The antithesis of this is when the competition trait is selfish, where higher competition leads to an increase in the mortality of other individuals. One possible mechanism of this could be where plants are able to, through toxins or other means, decrease the reproductive success of vegetation in the surrounding area. An example of auto-allelopathy can be found in white clover, where its presence has been shown to decrease the density of surrounding vegetation including itself [39] as well as alfalfa [40]. An interesting extension to this model then, would be to consider when competition is selfish as opposed to altruistic.

The main focus of this study has been on the evolution of the host in the presence of a pathogen with varying degrees of transmissibility or virulence. As such, the evolution of the pathogen has not been considered as has been for previous studies [41,42]. These show that if there is a link between transmission and virulence, then the pathogen evolves such that the combined transmission and virulence maximizes the basic reproductive number of the disease [43]. An interesting extension then, would be what strategies emerge in the presence of a pathogen that is co-evolving with the host.

One of the assumptions is the mechanism that transmits the effects of competition is spatially independent of the mechanisms that transmit the disease. This would be justified for direct-contact and root-affecting pathogens [44]. For example, wasting disease in seagrass has been shown to transmit primarily through leaf-to-leaf contact [35]. Although long-range disease transmission through rafting of infected host material has also been postulated for seagrass, to our knowledge it has never been demonstrated/observed. However, for wind- or water-borne pathogens, competition and disease transmission may not be spatially separate and the processes would need to be considered correlated.

Our results show a unique mechanism under which regular pattern formation can arise due to evolutionary pressure from a pathogen. We have shown that the ability of a species to self-organize into a regular geometry greatly affects its ability to regulate disease. Further, we have demonstrated that if such a mechanism is heritable then there is a sharp transition (akin to a second-order phase transition in physics), where one trait dominates over another. We have demonstrated how the presence of a highly transmissible pathogen can lead to regular pattern formation in a vegetation or sessile organism system.
